# Twin Peaks: A/H1N1 Pandemic Influenza Virus Infection and Vaccination in Norway, 2009–2010

**DOI:** 10.1371/journal.pone.0151575

**Published:** 2016-03-24

**Authors:** Thierry Van Effelterre, Gaël Dos Santos, Vivek Shinde

**Affiliations:** 1 Global Epidemiology, GSK Vaccines, Wavre, Belgium; 2 Business & Decision Life Sciences (on behalf of GSK Vaccines), Brussels, Belgium; 3 GSK Vaccines, King of Prussia, PA, United States of America; University of Hong Kong, HONG KONG

## Abstract

**Background:**

Vaccination campaigns against A/H1N1 2009 pandemic influenza virus (A/H1N1p) began in autumn 2009 in Europe, after the declaration of the pandemic at a global level. This study aimed to estimate the proportion of individuals vaccinated against A/H1N1p in Norway who were already infected (asymptomatically or symptomatically) by A/H1N1p before vaccination, using a mathematical model.

**Methods:**

A dynamic, mechanistic, mathematical model of A/H1N1p transmission was developed for the Norwegian population. The model parameters were estimated by calibrating the model-projected number of symptomatic A/H1N1p cases to the number of laboratory-confirmed A/H1N1p cases reported to the surveillance system, accounting for potential under-reporting. It was assumed in the base case that the likelihood of vaccination was independent of infection/disease state. A sensitivity analysis explored the effects of four scenarios in which current or previous symptomatic A/H1N1p infection would influence the likelihood of being vaccinated.

**Results:**

The number of model-projected symptomatic A/H1N1p cases by week during the epidemic, accounting for under-reporting and timing, closely matched that of the laboratory-confirmed A/H1N1p cases reported to the surveillance system. The model-projected incidence of symptomatic A/H1N1p infection was 27% overall, 55% in people <10 years old and 41% in people 10–20 years old. The model-projected percentage of individuals vaccinated against A/H1N1p who were already infected with A/H1N1p before being vaccinated was 56% overall, 62% in people <10 years old and 66% in people 10–20 years old. The results were sensitive to assumptions about the independence of vaccination and infection; however, even when current or previous symptomatic A/H1N1p infection was assumed to reduce the likelihood of vaccination, the estimated percentage of individuals who were infected before vaccination remained at least 32% in all age groups.

**Conclusion:**

This analysis suggests that over half the people vaccinated against A/H1N1p in Norway during the 2009 pandemic may already have been infected by A/H1N1p before being vaccinated.

## Introduction

The 2009 pandemic was caused by the emergence of influenza A(H1N1)pdm09 virus, a novel strain of influenza virus A(H1N1) with a unique combination of influenza viruses genes never previously detected in animals or humans. The first cases were detected in Mexico and California in spring 2009, and the World Health Organization declared a pandemic (phase 6) in June 2009 [1]. The first cases of A/H1N1 2009 pandemic influenza virus in Europe were reported in late April 2009 in travellers returning from Mexico, followed by an initial wave of local transmission in spring and summer 2009, outside the normal European influenza season, and a much larger wave of transmission in autumn and winter 2009 [2], reaching a peak at around week 48 (early December) 2009 [2]. The infection was most commonly detected in children aged <14 years, and many persons born before the mid-1950s had some level of immunity to the A/H1N1 2009 pandemic influenza virus as a result of exposure to a previous antigenically similar ancestor influenza virus [2].

Vaccines against the A/H1N1 2009 pandemic influenza virus were rapidly developed after declaration of Phase 6 of the pandemic. European marketing authorisation was granted for three vaccines: adjuvanted (AS03) A/H1N1 2009 pandemic influenza vaccine *(Pandemrix*^™^; *Pandemrix*^™^ is a trade mark of the GSK group of companies); another adjuvanted vaccine (*Focetria*^™^; *Focetria*^™^ is a trade mark of Novartis Vaccines and Diagnostics); and a non-adjuvanted vaccine (*Celvapan*^™^; *Celvapan*^™^ is a trade mark of Baxter AG) [2]. Vaccination coverage was highly variable between countries [2].

A safety signal indicating an association between narcolepsy cases in children and adolescents and vaccination with adjuvanted (AS03) A/H1N1 2009 pandemic influenza vaccine was reported in Sweden and Finland and became public in 2010 several months after the end of the vaccination campaigns in Europe [3]. Narcolepsy is a rare neurological disorder characterised by unintentional daytime lapses into sleep and cataplexy (sudden muscle weakness associated with emotions), with a typical age of onset of around age 12–16 years [4]. The incidence for narcolepsy with cataplexy has been estimated at 0.74 cases per 100,000 person-years and 1.37 per 100,000 person years for narcolepsy with or without cataplexy [5]. Incidence rates up to 3.84 per 100,000 person years have been reported in 10–19-year-olds in the United States of America (US) [5]. Associations between narcolepsy and adjuvanted (AS03) A/H1N1 2009 pandemic influenza vaccine were reported in children and adolescents in Sweden, Finland, Norway, Ireland, England and France, with narcolepsy incidence ranging from 4.2 per 100,000 person-years in Sweden to 10 per 100,000 person-years in Norway [6].

**Fig 1:**
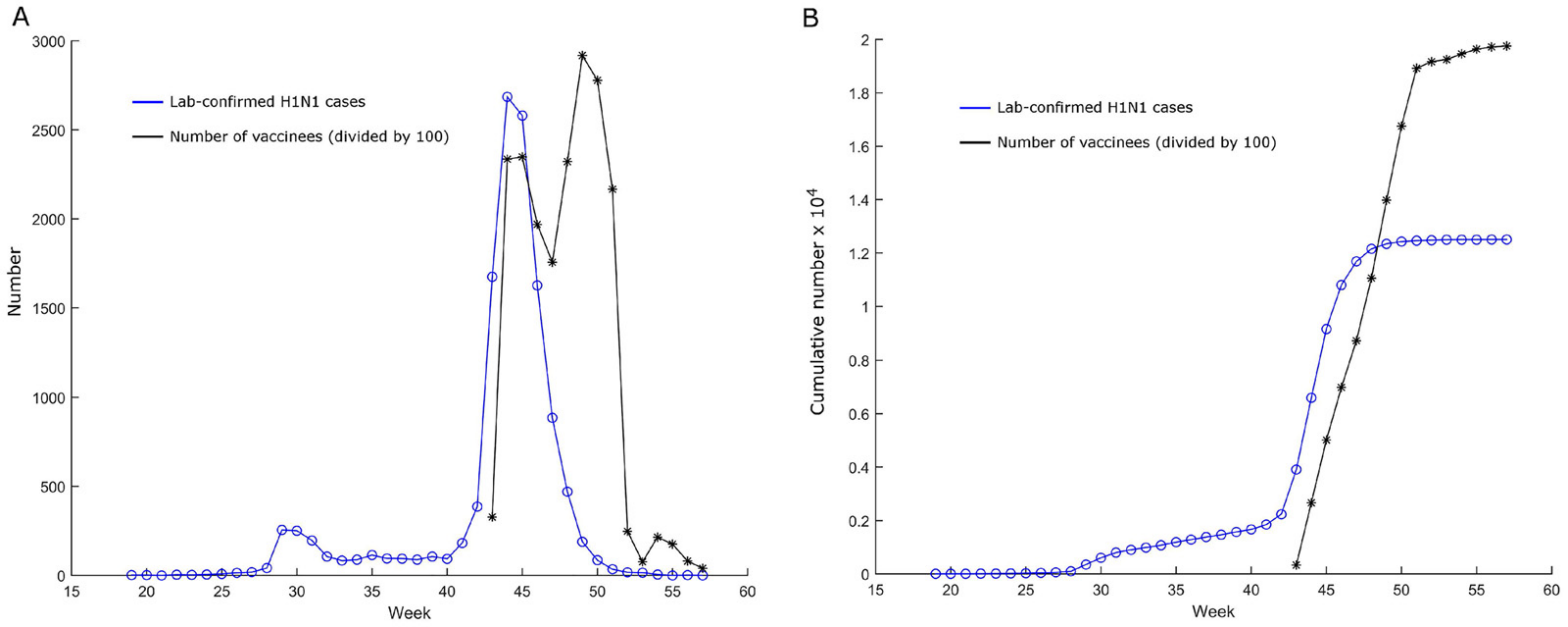
Temporal association between the A/H1N1 2009 influenza virus pandemic and vaccination programme in Norway. (A) Number of laboratory-confirmed cases of A/H1N1 2009 pandemic influenza virus reported through the surveillance system and number of A/H1N1 2009 pandemic influenza virus vaccinees divided by 100, by week of 2009–2010. (B) Cumulative number of laboratory-confirmed cases of A/H1N1 2009 pandemic influenza virus reported through the surveillance system and cumulative number of A/H1N1 2009 pandemic influenza virus vaccinees divided by 100. The first four weeks of 2010 are designated as weeks 54–57.

The cause of this observed association between narcolepsy and adjuvanted (AS03) A/H1N1 2009 pandemic influenza vaccine is not yet established. An increase in narcolepsy was observed following the A/H1N1 2009 influenza pandemic in China although the number of cases reporting a prior H1N1 vaccination was very low and only unadjuvanted vaccines were deployed, suggesting that narcolepsy could be related to natural A/H1N1 2009 pandemic influenza virus infection [7]. In Norway, delivery of adjuvanted (AS03) A/H1N1 2009 pandemic influenza vaccine began in October 2009 after the declaration of the A/H1N1 2009 influenza pandemic [8], and thus the pandemic and the programme of vaccination against A/H1N1 2009 pandemic influenza virus occurred concurrently, as illustrated in Fig 1. This raised the possibility that a substantial number of individuals vaccinated against A/H1N1 2009 pandemic influenza virus could have already been infected with A/H1N1 2009 pandemic influenza virus before being vaccinated.

Natural infection with A/H1N1 2009 pandemic influenza virus may be a potential causal factor in the development of narcolepsy, based on the observation of narcolepsy cases in China even though the number of vaccinees was low. Unfortunately, the ‘real’ proportion of individuals vaccinated against A/H1N1 2009 pandemic influenza virus who were already infected with A/H1N1 2009 pandemic influenza virus before vaccination is not currently known. The objective of the present model-based analysis was to estimate the proportion of individuals vaccinated against A/H1N1 2009 pandemic influenza virus in Norway during the 2009 pandemic who had already been infected with A/H1N1 2009 pandemic influenza virus (with or without symptoms) prior to receiving A/H1N1 2009 pandemic influenza virus vaccination, using a mathematical model of A/H1N1 2009 pandemic influenza virus transmission. Norway was selected for the analysis due to the availability of epidemiological and vaccination data for the A/H1N1 2009 influenza pandemic in addition to a high vaccination coverage rate. The A/H1N1 2009 influenza pandemic in Norway was epidemiologically similar to Sweden and Finland, countries where the signal of an association between adjuvanted (AS03) A/H1N1 2009 pandemic influenza vaccine and narcolepsy first appeared. In addition, both countries reported the largest number of vaccine-associated narcolepsy cases. Quantitative information on the number of vaccinees who were already infected with A/H1N1 2009 pandemic influenza virus before receiving A/H1N1 2009 pandemic influenza virus vaccination in Europe may inform a better understanding of the relationship between vaccination, natural A/H1N1 2009 pandemic influenza virus infection and narcolepsy.

## Materials and Methods

### Input data

The population modelled included all individuals aged 0–86 years in Norway, representing 98% of the total population in 2009. Demographic data on the Norwegian population, stratified into eight 10-year age groups up to age 80 years and a single age group for age 80+ years, were obtained from the US Census Bureau International Database [9]. The total population of Norway in 2009 was 4,830,658 [9].

Data on the contact patterns between different age groups were taken from the POLYMOD study in neighbouring Finland [10], since country-specific contact data for Norway were not available, and was recalculated using the method [11]. An adjustment factor (constrained to be between 0 and 1) for the groups aged 0–20 years was included in the model in the calibration process, so that the model fitting would not be excessively sensitive to the probability of contact in that age group.

Data on the number of laboratory-confirmed A/H1N1 2009 pandemic influenza virus cases reported to the surveillance system in Norway during the 2009 pandemic by week, stratified into eight 10-year age groups up to age 80 years and a single age group for age 80+ years, were obtained from the Norway Public Health Institute.

Data on the total number of individuals vaccinated with adjuvanted (AS03) A/H1N1 2009 pandemic influenza vaccine during the 2009 pandemic by week, stratified into eight 10-year age groups up to age 80 years and a single age group for age 80+ years, were obtained from the Norway Public Health Institute. Table 1 shows the percentage of each age group and of the overall population vaccinated during the 2009 pandemic in Norway.

**Table 1:** Percentage of individuals in Norway vaccinated with adjuvanted (AS03) A/H1N1 2009 pandemic influenza vaccine during the A/H1N1 2009 influenza virus pandemic.

Age group (years)	Percentage vaccinated
0–<10	60%
10–<20	44%
20–<30	27%
30–<50	38%
50+	46%
**Overall**	**43%**

### Model structure

The model was a mechanistic dynamic model of A/H1N1 2009 pandemic influenza virus transmission. Modelled outcomes included the total projected incidence of symptomatic A/H1N1 2009 pandemic influenza cases as a percentage of the population, the percentage of the vaccinated population who were already infected at the time of vaccination, and the percentage of the population in each of four categories of vaccination/infection status at the end of the pandemic:

- Not vaccinated and not infected;
- Not vaccinated and infected;
- Vaccinated and not infected;
- Vaccinated and infected, also with the subset of those vaccinated and infected by A/H1N1 2009 pandemic influenza virus before vaccination.

These outcomes were projected for five age groups (0–<10 years, 10–<20 years, 20–<30 years, 30–<50 years and 50+ years) and overall. The model was also used to estimate the value of the *basic reproduction number (R*_*0*_*)* for A/H1N1 2009 pandemic influenza virus in Norway during the 2009–2010 pandemic.

The model structure is shown in S1 Fig. The population aged 0–86 years was stratified into 1-year age groups, and within each age group people were stratified into compartments according to vaccination status and A/H1N1 2009 pandemic influenza virus infection/disease status. Non-vaccinated individuals flowed over time between these compartments, from Susceptible to Latent (infected but not yet infectious) when infected with A/H1N1 2009 pandemic influenza virus, then to Infectious (infected and infectious), and finally to Removed after recovery from the infection. Infectious individuals were divided between symptomatic and asymptomatic, and the percentage who were symptomatic was modelled as an exponentially decreasing function of age, with the rate of decrease, *r*, and the value in the youngest age group, *H*, estimated by calibration of the model to the data on the number of laboratory-confirmed A/H1N1 2009 pandemic influenza cases reported. Recovered individuals were also divided between Recovered after symptomatic and asymptomatic infection.

The mean duration of latency was assumed to be 1.2 days, and the mean duration of the Infectious state was assumed to be 5.6 days. In the model, the Latent state was divided into three sub-states of equal duration, and the Infectious states were each divided into five sub-states of equal duration. Infectiousness was assumed to be maximal in the first two states, then half this value in the next two states, then one-quarter of the maximal value in the last state. Asymptomatic individuals were assumed to be less infectious than symptomatic individuals by a factor of *f*, whose value was estimated by calibration. These same model compartments were replicated for the vaccinated individuals with specific states during the first week post-vaccination (during which the vaccine was assumed to have no effect yet) and from 1 week post-vaccination onwards (when the vaccine was assumed to be effective).

The time horizon of the model was the duration of the 2009 pandemic in Norway. As this was a short period of less than one year, the population was assumed to be constant and there were no demographic in/out flows or flows between the age groups.

Individuals in each infection/disease state flowed from the unvaccinated to the corresponding vaccinated state in discrete 1-week time steps. The number of individuals vaccinated was taken from the weekly number of vaccinated individuals by 10-year age group up to 80 years of age and in the 80+ years-old. This was multiplied by the fraction of population in that specific 1-year age group out of the population in each corresponding larger age group to obtain the number for each 1-year age group. In the base case, vaccination was assumed to be independent of the infection/disease state. The sensitivity of the model outcomes to that assumption was evaluated using four different scenarios (see below).

The *force of infection* (e.g. the per-susceptible risk of infection) in a given age group depends on the contact rate between this age group and other age groups, the number of infectious individuals in each age group, the transmissibility parameter *t*, and the infectiousness in each of the Infectious sub-states. The *force of infection* in vaccinated individuals is given by the *force of infection* in non-vaccinated individuals multiplied by 1 minus the vaccine efficacy.

Usually, laboratory-confirmed A/H1N1 2009 pandemic influenza virus infections represent only a fraction of total A/H1N1 2009 pandemic influenza virus symptomatic infections. To account for potential under-reporting of symptomatic infection, the model adjusted for this using age-group-specific under-reporting factors for each of six age groups (0–<10 years, 10–<20 years, 20–<30 years, 30–<40 years, 40–<50 years and 50+ years). These were estimated by minimising the sum of squares of differences between the number of symptomatic A/H1N1 2009 pandemic influenza cases projected by the model and the number of laboratory-confirmed A/H1N1 2009 pandemic influenza cases reported by the surveillance system, accounting for the estimated under-reporting factors. A delay of 1 day between the development of symptoms and reporting of the case was assumed in the model.

Susceptibility to the A/H1N1 2009 pandemic influenza virus was set at 100% in the population aged <60 years. In the population aged 60+ years, the percentage of people with pre-existing immunity to the virus (who were assumed in the model to be in the Recovered after asymptomatic infection state at the beginning of the 2009 H1N1 epidemic) was assumed to increase linearly with age, from 16% at age 61 years to 25% at age 86 years [12]. All the percentages of individuals infected prior to vaccination presented in the Results section include this small percentage of individuals assumed to be immune prior to the start of the 2009 pandemic.

The value of the *basic reproduction number* (R_0_) for the A/H1N1 2009 pandemic influenza virus in Norway during the 2009–2010 pandemic was estimated using the next generation matrix [13].

The efficacy of adjuvanted (AS03) A/H1N1 2009 pandemic influenza vaccine was set at 90% in individuals aged up to 65 years and 80% in those aged 66 years or more. Other articles referring to vaccine effectiveness have reported similar numbers [14]. The vaccine effect was modelled as a reduction in the risk of A/H1N1 2009 pandemic influenza virus infection in vaccinated individuals. The vaccine was assumed to be effective 7 days after administration.

### Model fitting

A number of model parameters needed to be estimated from the data available. These parameters were estimated by attempting to reproduce the reported number of laboratory-confirmed cases over time as well as possible, accounting for both under-reporting and the percentage of cases that are symptomatic by age. The relationship between the number of laboratory-confirmed cases reported through the surveillance system and the ‘true’ number of symptomatic infections is not straightforward, as both the under-reporting factor and the percentage of cases that are symptomatic may vary by age. Six model parameters (*s*, *t*, *H*, *r*, *f* and *a*) were estimated by calibration (Table 2).

**Table 2:** Model parameters estimated by calibration.

Parameter	Description
s	Starting time of pandemic in days (day 0 = first day of 2009—week 1)
*t*	Transmissibility parameter
*H*	Percentage (%) of A/H1N1 2009 pandemic influenza virus infections that are symptomatic in the youngest age group
*r*	Rate of exponential decay of the percentage of infections that are symptomatic as a function of age (unit: 1/year)
*f*	Factor for the relative infectiousness of asymptomatic compared with symptomatic individuals
*a*	Factor for the POLYMOD contact matrix between susceptible individuals aged 0–<20 years and infectious individuals aged 0-<20 years

The percentage of infections that were symptomatic was constrained to be at least 25% at any age, and 30–40% at age 50 years [15]. For a given set of values of these 6 model parameters (*s*, *t*, *H*, *r*, *f* and *a*), the six age-group-specific under-reporting factors *u*_*j*_ were derived by minimizing *Σ*_*w*_ *Σ*_*j*_ *(O*_*wj*_*− (f*_*j*_ *x M*_*wj*_*))* ^*2*^ with respect to *f*_*1*_, *f*_*2*_, *…*, *f*_6,_ giving the estimates *u*_*j*_ = *1/f*_*j*_ = *(Σ*_*w*_ *M*_*wj*_^*2*^*) / (Σ*_*w*_ *O*_*wj*_ *x M*_*wj*_*)*. The six model parameters (*s*, *t*, *H*, *r*, *f* and *a*) were estimated by minimizing the total sum of squares of the differences between the number of laboratory-confirmed A/H1N1 2009 pandemic influenza cases reported by surveillance and the number of symptomatic A/H1N1 cases projected by the model for the corresponding week/age group, accounting for (e.g. down-scaled) the estimated age-group-specific under-reporting factors as derived above. More precisely, the objective function (OBJ) that was minimized is

$$OBJ\;=\;\Sigma_{w}\Sigma_{j}{\left(O_{wj}-\;\left(M_{wj}\left(s,\;t,\;H,\;r,\;f,\;a\right)/u_{j}\right)\;\right)}^{2},$$

where *O*_*wj*_ is the number of laboratory-confirmed A/H1N1 2009 pandemic influenza cases reported by surveillance in age group *j* and week *w*, *M*_*wj*_ is the number of symptomatic A/H1N1 cases projected by the model in week *w* and age group *j*, and *u*_*j*_ is the estimated under-reporting factor in age group *j*, with the index on *w* running from w = 2009-week 41 to w = 2010-week 4, and the index for *j* running from 1 to 6 (6 age groups: 0-<10, 10-<20, 20-<30, 30-<40, 40-<50 and 50+ years). The objective function *OBJ* was minimized using a simplex search method without constrains (using the *Matlab* function *fminsearch*). In the minimization process, the parameters were constrained to lie between the corresponding lower and upper bounds (see S1 Table) by penalizing the objective function for values outside those bounds.

The model was developed in *Matlab* (MathWorks, Inc., version 2015a).

### Sensitivity analysis

The sensitivity of the model to the assumption that vaccination was independent of the infection/disease state was tested by calibrating and running the model for four additional scenarios. These scenarios were as follows (the base case was designated Scenario 1):

- Scenario 2. Current symptomatic infection makes vaccination more likely (as many individuals as possible currently in the “Infectious symptomatic” state are vaccinated);
- Scenario 3: Current or previous symptomatic infection makes vaccination more likely (as many individuals as possible currently in the “Infectious symptomatic” or “Recovered after symptomatic” states are vaccinated);
- Scenario 4: Current symptomatic infection makes vaccination less likely (as many individuals as possible currently in states other than the “Infectious symptomatic” state are vaccinated);
- Scenario 5: Current or previous symptomatic infection makes vaccination less likely (as many individuals as possible currently in states other than the “Infectious symptomatic” or “Recovered after symptomatic” states are vaccinated).

## Results

### Model fitting

**Fig 2:**
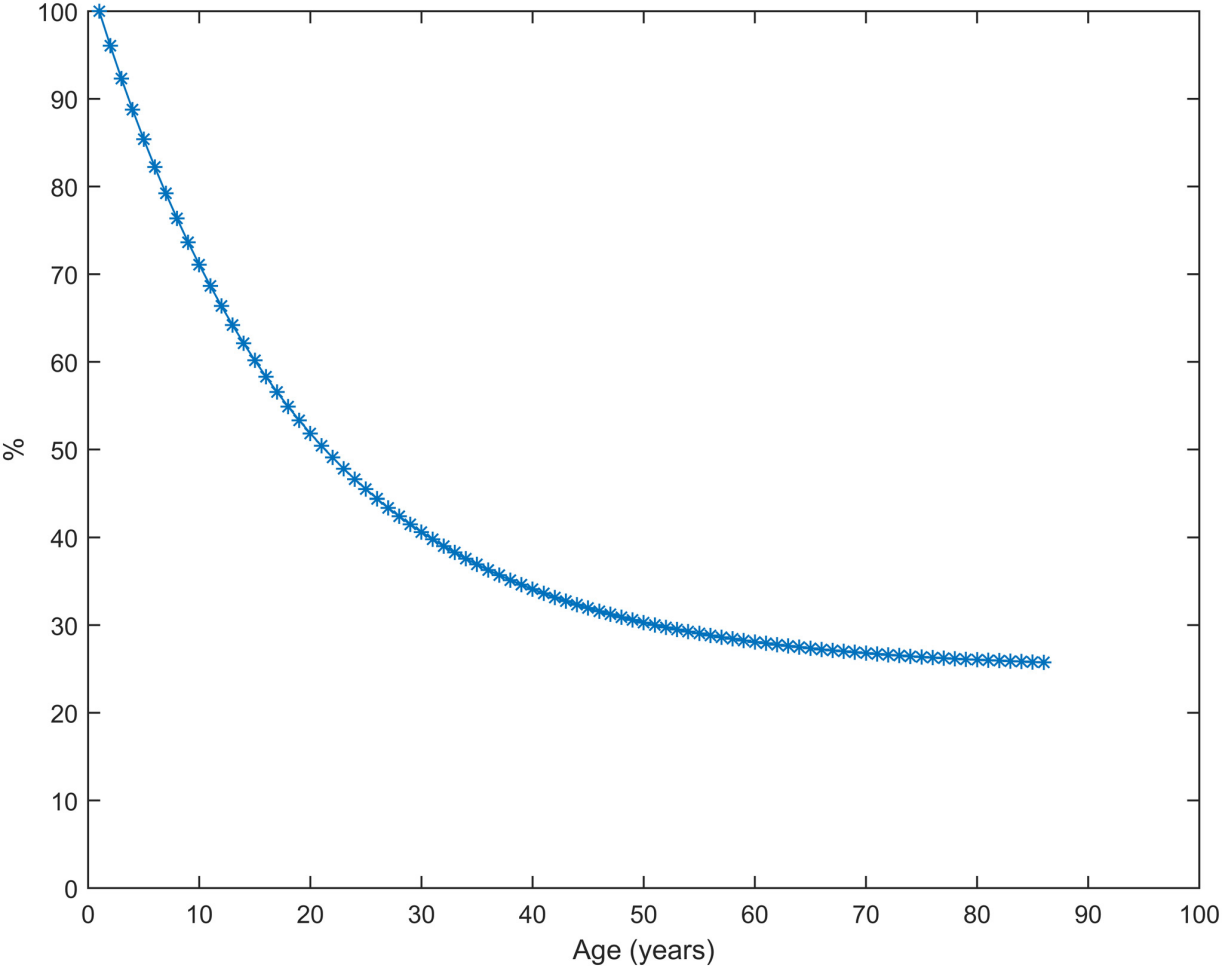
Estimated percentage of A/H1N1 2009 pandemic influenza infections that are symptomatic, by age. Derived from the fitted parameters *H* and *r*.

The best fit values for the six calibrated parameters are shown in S1 Table. Fig 2 shows the estimated age-specific percentage of infections that are symptomatic, and Table 3 shows the six estimated age-specific under-reporting factors.

**Table 3:** Estimated age-specific under-reporting factors.

Age group (years)	Estimated age-group-specific under-reporting factor*
0 -<10	105.13
10 -<20	91.97
20 -<30	148.40
30 -<40	154.47
40 -<50	181.68
50+	216.11

* For example a factor of 105.13 indicates that there were 105.13 times more symptomatic cases than were reported.

### Model projections vs. reported laboratory-confirmed cases

**Fig 3:**
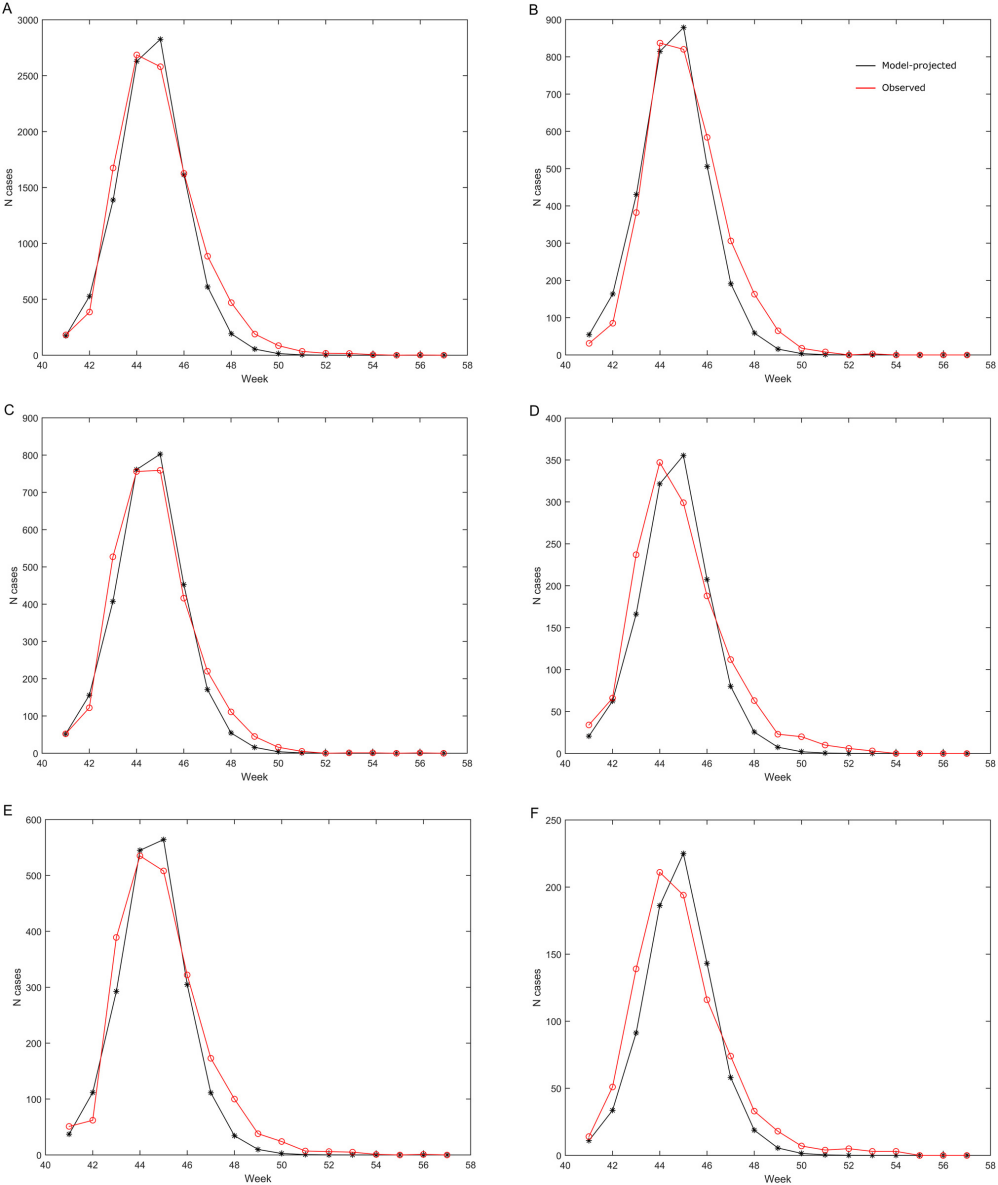
Number of symptomatic A/H1N1 2009 pandemic influenza cases projected by the model compared with the observed number of laboratory-confirmed A/H1N1 2009 pandemic influenza cases. (A) all ages; (B) age 0–<10 years; (C) age 10–<20 years; (D) age 20–<30 years; (E) age 30–<50 years (pooled); (F) age 50+ years. Black: Model-projected cases scaled down to account for age-specific under-reporting factors. Red: Observed laboratory-confirmed cases reported to the surveillance system. Week number refers to 2009, and the first four weeks of 2010 are designated as weeks 54–57.

The number of symptomatic A/H1N1 2009 pandemic influenza cases by week projected by the model matched well with the number of laboratory-confirmed A/H1N1 2009 pandemic influenza cases reported through the surveillance system in all age groups combined (Fig 3A) and in individual age groups (Fig 3B–3F).

### Model-projected incidence of symptomatic A/H1N1 2009 pandemic influenza virus infection

Table 4 shows the model-projected incidence of symptomatic A/H1N1 2009 pandemic influenza virus infection in Norway during the 2009 pandemic for the base case by age and pooled across all age groups. The incidence was highest in the two youngest age groups, at 55% in the group aged <10 years and 41% in the group aged 10–<20 years.

**Table 4:** Incidence of symptomatic A/H1N1 2009 pandemic influenza virus infection and percentage of vaccinated individuals already infected before vaccination in Norway during the 2009 pandemic. Middle column: Model-projected percentage with symptomatic A/H1N1 2009 pandemic influenza virus infection, by age and pooled across all age groups. Right column: Model-projected percentage of vaccinees at the end of the pandemic who were infected with A/H1N1 2009 pandemic influenza virus before receiving vaccination in Norway, by age and pooled across all age groups. Base case.

Age group (years)	Percentage of age group or all with symptomatic A/H1N1 2009 pandemic influenza virus infection	Percentage of vaccinated individuals who were already infected with A/H1N1 2009 pandemic influenza virus before receiving vaccination
0–<10	55%	62%
10–<20	41%	66%
20–<30	31%	59%
30–<50	25%	63%
50+	11%	44%
All	27%	56%

### Infection status at the time of vaccination

Table 4 also shows the model-based estimates of the percentage of the vaccinated population at the end of the pandemic who were infected with A/H1N1 2009 pandemic influenza virus before receiving A/H1N1 2009 pandemic influenza virus vaccination, i.e. people in the Latent, Infectious and Recovered states at the time of vaccination. By the end of the pandemic, the model estimated that the percentage of vaccinated individuals who were already infected with the A/H1N1 2009 pandemic influenza virus before vaccination was 62% in the group aged <10 years old, 66% in the group aged 10–20 years old and 56% overall (Table 4).

**Fig 4:**
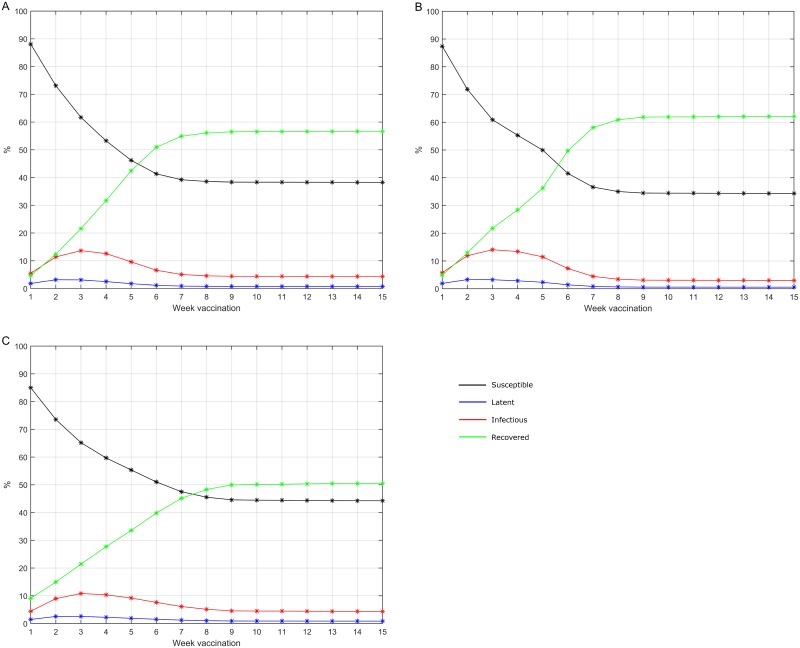
A/H1N1 2009 pandemic influenza virus infection status at H1N1 vaccination, over time. Model-projected percentage of the cumulative number vaccinated. (A) age 0–<10 years; (B) age 10–<20 years; (C) all age groups.

Fig 4 shows the model projections for the proportion of vaccinated individuals by state at the time of vaccination: Susceptible, Latent, Infectious (asymptomatic or symptomatic) and Recovered (after asymptomatic or after symptomatic infection). All percentages are presented cumulatively over time as a percentage of the vaccinated population (in the age group or overall).

The percentage of the population in each of the four vaccination/infection status categories at the end of the pandemic is shown for each age group and the overall population in S2 Table.

### Basic reproduction number

The value of the *basic reproduction number (R*_*0*_*)* for the A/H1N1 2009 pandemic influenza virus in Norway during the 2009–2010 pandemic was estimated to be 1.8.

### Sensitivity analysis

Table 5 also shows the model outcomes for the four additional scenarios exploring the effect of different assumptions about the relationship between vaccination and infection. The base case (Scenario 1) assumed that vaccination was independent of the infection/disease status and is shown for comparison.

**Table 5:** Sensitivity analysis. Model-projected percentage of vaccinees at the end of the pandemic who were infected with A/H1N1 2009 pandemic influenza virus before receiving vaccination in Norway, by age, for each of the scenarios tested.

	Percentage of vaccinees infected with A/H1N1 2009 pandemic influenza virus before receiving A/H1N1 2009 pandemic influenza virus vaccination					
Age group (years)	Base case (Scenario 1)	Scenario 2^a^	Scenario 3 ^a^	Scenario 4 ^a^	Scenario 5 ^a^	Immune at start *
0–<10	62%	74%	100%	60%	32%	0%
10–<20	66%	76%	96%	65%	45%	0%
20–<30	59%	84%	100%	57%	45%	0%
30–<50	63%	82%	90%	62%	54%	0%
50+	44%	51%	55%	43%	39%	12%
50-<65**	48%	59%	65%	47%	41%	5%
65+**	38%	42%	43%	38%	35%	21%
All	56%	69%	81%	55%	43%	4%

^**a**^ See definition of the four scenarios in the Materials and Methods section.

*age-group-specific percentage assumed immune at start of the H1N1 epidemic.

** outcome in the 50+ are also presented splited 50-<65 and 65+

In the scenarios in which (respectively current or previous and current) symptomatic infection was assumed to make vaccination more likely (Scenarios 2 and 3, respectively), the percentage of individuals projected to have been infected before vaccination was higher than in the base case, up to 100% in the groups aged 0-<10 and 20–<30 years. Conversely, in the scenarios in which (respectively current or previous and current) symptomatic infection was assumed to make vaccination less likely (Scenarios 4 and 5, respectively), the percentage infected before vaccination was lower than the base case, as would be expected. However, even in these scenarios, the percentage of individuals projected to have been infected before vaccination never fell below 32% in any age group.

The value of the *basic reproduction number (R*_*0*_*)* estimated from the model was approximately the same in the 4 scenarios of the sensitivity analysis as for the base case.

S2 Table shows the model projections for the percentage of the population in each of the vaccination/infection categories at the end of the A/H1N1 2009 influenza pandemic in Norway, for each of the four scenarios.

## Discussion

This dynamic mathematical model aimed to quantify the percentage of the A/H1N1-vaccinated population in Norway who had already been infected with A/H1N1 2009 pandemic influenza virus before receiving vaccination during the mass vaccination campaign against A/H1N1 2009 pandemic influenza. The close temporal association between the pandemic and the vaccination programme during the autumn and winter of 2009 is well known, and this makes it likely that a substantial number of people would have already been infected before they were vaccinated. However, to our knowledge this is the first analysis aiming to quantify through mathematical modelling the percentage of vaccinees who had already been infected by A/H1N1 2009 pandemic influenza virus before being vaccinated.

Our estimates of the attack rate (incidence during the pandemic) of A/H1N1 2009 pandemic influenza virus results are broadly consistent with the findings of an earlier mathematical model of the A/H1N1 2009 influenza pandemic in Norway, published in 2012 by Freiesleben de Blasio and colleagues [8]. The earlier model projected an overall attack rate of approximately 30%, with the highest rate in the youngest age group (43–44% in the group aged 0–14 years) [8]. In the present analysis the projected attack rate was slightly lower, at 27% overall, and the highest projected incidence of symptomatic A/H1N1 2009 pandemic influenza virus infection was also in the youngest age group modelled, aged 0–<10 years (55%).

The objective of the model of Freiesleben de Blasio et al. differed from ours, as it aimed to evaluate the impact of vaccination and antiviral treatment on the progress of the A/H1N1 2009 influenza virus pandemic and to explore the potential effects of beginning the vaccination programme earlier or later. Most of the assumptions about the natural history of A/H1N1 2009 pandemic influenza virus transmission are similar in both models. Our model differs from that of Freiesleben de Blasio et al. on four main aspects. First, we calibrated our model on the observed numbers of laboratory-confirmed A/H1N1 2009 pandemic influenza cases in Norway, whereas Freiesleben de Blasio et al. calibrated their model on cases of influenza-like illness. Second, the present model is more finely stratified with respect to age than the earlier model, using six age groups instead of three in the Freiesleben de Blasio model. Third, we used values of 1.2 days for the latency period and 5.6 days for the infectious period, compared with values of 1.9 days for the incubation period (with some low infectiousness during the final period accounting for 1/3 of the incubation period) and 5.0 days for symptomatic and asymptomatic infections, used by Freiesleben de Blasio et al. Finally, Freiesleben de Blasio et al. assumed that a fixed percentage of cases were symptomatic (65% in individuals aged 0–14 years and 55% in individuals aged 15+ years), whereas in the present model the percentage of cases that were symptomatic was estimated by calibration as a decreasing function of age to better account for age specificity associated with the disease.

In the absence of data, we assumed for the base case that vaccination against A/H1N1 2009 pandemic influenza virus was independent of A/H1N1 2009 pandemic influenza virus infection/disease status. This is a key assumption in the analysis. To explore its effect, we tested four alternative scenarios in the sensitivity analysis, in which current or previous and current A/H1N1 2009 pandemic influenza virus symptomatic infection made vaccination more (Scenarios 2 and 3) or less (Scenarios 4 and 5) likely. The results were sensitive to changes in the assumption of independence, as would be expected. We also tested the sensitivity of the model outcomes to the frequency at which individuals in each infection/disease state flowed from the unvaccinated to the corresponding vaccinated state, using 3 transitions per week instead of 1 (with the number of vaccinated individuals divided by 3). This analysis (not presented) indicated that changes in this assumption had relatively little effect on the results.

The value of 1.8 for the *basic reproduction number (R*_*0*_*)* estimated from the model was somewhat greater than estimated by Freiesleben de Blasio et al. (1.37–1.39) [8]. This may be related to the fact that Freiesleben de Blasio et al. used influenza-like illness for model calibration while the model presented here used laboratory-confirmed cases. However, it is noteworthy that both estimates are of a similar order of magnitude, despite the slight differences between the analyses outlined above.

According to published data, a sizable proportion of older adults were protected by prior exposure to a similar influenza virus that had been circulating before the mid-1950s, with a higher proportion of pre-existing cross-reactive functional antibodies capable of neutralizing A/H1N1 2009, among the older birth cohorts [12,16].

This finding supports our approach to consider that the percentage of individuals with pre-existing immunity increases with age in subjects above 60 years of age.

There are many uncertainties about the natural history of A/H1N1 2009 pandemic influenza virus, including the percentage of infections that are symptomatic and the degree of under-reporting of symptomatic cases. In the present model we had to estimate these parameters by calibration to reported epidemiological data, and this is a limitation of our analysis.

A recent paper aiming at assessing the prevalence of antibodies reactive to the 2009 pandemic influenza A(H1N1) after the main epidemic wave reported figures overall consistent with our estimates obtain thanks to the model-projected percentage with both symptomatic and asymptomatic A/H1N1 2009 pandemic influenza virus infection (S2 Table) with slight variability related to the HI threshold considered (HI titre ≥20 or HI titre ≥40) [17]. Estimates however differed for subjects between 20 and 50 years of age. This difference could be attributable to the different approach used to account for exposure to the virus. In our model, we considered the observed numbers of laboratory-confirmed A/H1N1 2009 pandemic influenza cases in Norway to which we applied the estimated age-specific under-reporting factors. Waalen et al., used serum antibody titres, determined by hemagglutination–inhibition (HI) test to evaluate the prevalence of antibodies to the 2009 pandemic influenza A(H1N1) virus. In the study, the vaccination status of the serum donors was not known, which precluded differentiating between seropositivity resulting from infection, from immunization, or from a combination of the two which limit any direct comparison with our findings.

Despite extensive research on the topic, the mechanisms underlying the association between the adjuvanted (AS03) A/H1N1 2009 pandemic influenza vaccine and the occurrence of narcolepsy remain unclear [3,18] with several challenges to account for all counfounders and potential biases [19]. A retrospective study in China found that narcolepsy onset was associated with a strong seasonality, and narcolepsy incidence increased by three-fold following the A/H1N1 2009 influenza pandemic [7]. In this study, only a very small fraction of subjects diagnosed in 2010 (5.6%) reported a prior vaccination against pH1N1, suggesting that pH1N1 unadjuvanted monovalent vaccination was not the trigger for increased narcolepsy onsets in China [7]. Interestingly, Han et al. found that a delay between infection and onset was around 6 months and the increase disappeared 2 years after the 2009 H1N1 winter flu pandemic [20]. Supporting this hypothesis, a recent paper in mice has demonstrated that the H1N1 influenza virus induces narcolepsy-like sleep disruption. According to the researchers, because noticeable changes occurred in the absence of adaptive autoimmune responses, it further emphasizes that brain infections with H1N1 virus have the potential to induce narcoleptic-like sleep disruption [21].

In addition, the authors of a recent retrospective epidemiological study coordinated by the Paul-Ehrlich Institut (PEI) have reported that despite the low pH1N1 vaccine coverage in Germany (<8.1% in any of the age segments), in individuals under 18 years of age, the incidence rates continuously increased from spring 2009 [22]. They further conclude that considering the low vaccination coverage in Germany during the 2009 pandemic, the significant increase of the incidence rate of narcolepsy observed in children and adolescents in the pandemic period (during and post mass vaccination campaign and in the post-pandemic period) as compared to the reference period (2.4-fold and 3.6-fold increase, respectively) may be attributable to other causes than the vaccination.

Narcolepsy is strongly associated with human leukocyte antigen class II genetic markers, and a leading hypothesis for its cause is that it is likely to be an autoimmune disorder resulting in the destruction of hypocretin-producing neurones in the hypothalamus [4]. Autoimmune diseases are considered to be multifactorial, with several environmental triggers contributing to the disease process [4]. Exposure to the A/H1N1 2009 pandemic influenza virus antigen during natural A/H1N1 2009 pandemic influenza virus infection has been hypothesized to be one potential trigger for the development of autoimmunity [4].

The present analysis provides model-based estimates of the percentage of the H1N1-vaccinated population in Norway who were infected with A/H1N1 2009 pandemic influenza virus before receiving vaccination. It will be helpful in informing the understanding of the relationships between vaccination, natural A/H1N1 2009 pandemic influenza virus infection and narcolepsy.

## Conclusions

The projections from this dynamic mathematical model of A/H1N1 2009 pandemic influenza virus transmission in Norway during the A/H1N1 2009 influenza pandemic estimated that 56% of individuals vaccinated against A/H1N1 2009 pandemic influenza virus (across all age groups) were infected with A/H1N1 2009 pandemic influenza virus before receiving vaccination when assuming independence between vaccination and infection status. This percentage was estimated to be 43% when current or previous symptomatic infection was assumed to make vaccination less likely. This model further supports the hypothesis that the vaccination against pH1N1 occurred slightly too late. Accumulative evidences suggest that the pandemic influenza A(H1N1) 2009 virus started circulating in Europe around week 16 of 2009 (with a declared worldwide pandemic outbreak of influenza at an international level, its highest point in week 23 [23]) whereas the mass vaccination campaign started in most of EU countries in week 39 onwards. This particular situation objectively precludes differentiating seasonal peaks in background illness from vaccine-induced effects and thus renders a rigorous assessment of the association between *Pandemrix*^*TM*^ and Narcolepsy very challenging [24].

This model-based quantitative evaluation gives some indication that a substantial fraction of individuals may have been infected by A/H1N1 2009 pandemic influenza virus prior to being vaccinated. This in turn may help to further clarify the association between A/H1N1 2009 pandemic influenza virus, vaccination and the development of narcolepsy.

### Trademark statement

*Pandemrix*^™^ is a trademark of the GSK group of companies. *Focetria*^™^ is a trademark of Novartis Vaccines and Diagnostics. *Celvapan*^™^ is a trademark of Baxter AG.

## Supporting Information

S1 Fig Model structure. Compartments and flows in the transmission model. There are similar compartments and flows for each of the 86 1-year age groups.
(TIF)

S1 Table Best fit model parameters estimated by calibration. (DOC)

S2 Table Model-projected status of the population at the end of the A/H1N1 2009 influenza virus pandemic in Norway. Percentage of the population in each of the following states at the end of the pandemic: not vaccinated and not infected; not vaccinated and infected; vaccinated and not infected; vaccinated and infected. Results shown by age and for the base case and each of the four scenarios tested.
(DOC)
