# Supplementary figures and images for: Twin Peaks: A/H1N1 Pandemic Influenza Virus Infection and Vaccination in Norway, 2009–2010
Source: PLoS One. 2016 Mar 24;11(3):e0151575. doi: 10.1371/journal.pone.0151575 (PMC4807012; doi:10.1371/journal.pone.0151575)

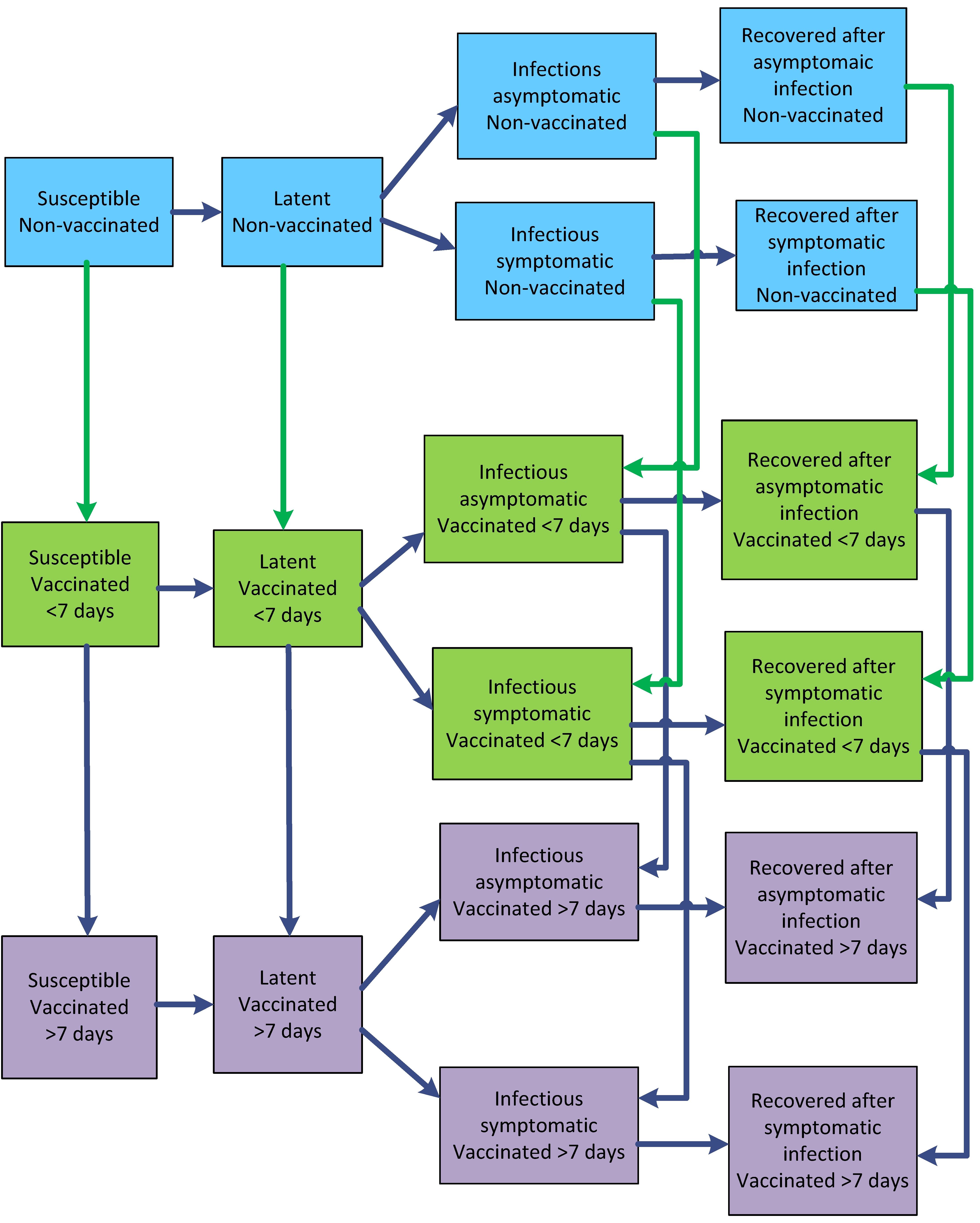

Supplement: S1 Fig — Compartments and flows in the transmission model. There are similar compartments and flows for each of the 86 1-year age groups. (TIF) [file pone.0151575.s001.tif]
