# Supplementary material for: Twin Peaks: A/H1N1 Pandemic Influenza Virus Infection and Vaccination in Norway, 2009–2010
Source: PLoS One. 2016 Mar 24;11(3):e0151575. doi: 10.1371/journal.pone.0151575 (PMC4807012; doi:10.1371/journal.pone.0151575)
Supplement: S1 Table — (DOC) [file pone.0151575.s002.doc]

S1 Table. Best fit model parameters obtained by calibration.

| **Parameter** | **Range for calibration** | | **Best fit** |
| --- | --- | --- | --- |
|  | **Lower bound** | **Upper bound** | **Base case** |
| Starting time of epidemic (days) ^a^ | 210 | 280 | 247.8125 |
| Transmissibility parameter, *t* | 0.02 | 0.12 | 0.0505 |
| Percentage of infections that are symptomatic in youngest age group, ­*H* | 0.95 | 1 | 1 |
| Rate of exponential decay of the percentage of infections that are symptomatic as a function of age, *r* (1/year) | 0.04 | 0.07 | 0.0541 |
| Factor for relative infectiousness of asymptomatic case compared with symptomatic case, *f* | 0.5 | 0.875 | 0.875 |
| Factor *a* for Polymod contact matrix in group aged 0–<20 years | 0.75 | 1 | 0.7731 |

^a^ Day 0 = first day of 2009 week 1
