# Supplementary material for: Twin Peaks: A/H1N1 Pandemic Influenza Virus Infection and Vaccination in Norway, 2009–2010
Source: PLoS One. 2016 Mar 24;11(3):e0151575. doi: 10.1371/journal.pone.0151575 (PMC4807012; doi:10.1371/journal.pone.0151575)
Supplement: S2 Table — Percentage of the population in each of the following states at the end of the pandemic: not vaccinated and not infected; not vaccinated and infected; vaccinated and not infected; vaccinated and infected. Results shown by age and for the base case and each of the four scenarios tested. (DOC) [file pone.0151575.s003.doc]

S2 Table. Model-projected status of the population at end of the A/H1N1 2009 influenza virus pandemic in Norway. Percentage of the population in each of the following states at the end of the pandemic: not vaccinated and not infected; not vaccinated and infected; vaccinated and not infected; vaccinated and infected. Results shown by age and for the base case and each of the four scenarios tested.

|  | |  |  | |  | | **Percentage in state (%)** | | | | | |
| --- | --- | --- | --- | --- | --- | --- | --- | --- | --- | --- | --- | --- |
| **Status at end of pandemic** | |  | **Age group (years)** | | | | | | |  |  | **All** |
| **Vaccination** | **Infection** | **Scenario** | **0–<10** | **10–<20** | | **20–<30** | | **30–<50** | **50+** | **50-<65*** | **65+*** |  |
| Not vaccinated | Not infected during pandemic | Base case | 15 | 17 | | 23 | | 17 | 25 | 23 | 28 | 20 |
|  |  | Scenario 2 | 17 | 18 | | 25 | | 19 | 26 | 25 | 28 | 22 |
|  |  | Scenario 3 | 31 | 27 | | 29 | | 21 | 27 | 26 | 29 | 26 |
|  |  | Scenario 4 | 14 | 17 | | 23 | | 16 | 25 | 23 | 27 | 20 |
|  |  | Scenario 5 | -0 | 9 | | 20 | | 14 | 23 | 21 | 26 | 15 |
| Not vaccinated | Infected during pandemic | Base case | 30 | 40 | | 52 | | 47 | 28 | 33 | 21 | 38 |
|  |  | Scenario 2 | 27 | 39 | | 50 | | 45 | 27 | 31 | 21 | 37 |
|  |  | Scenario 3 | 13 | 30 | | 46 | | 42 | 26 | 30 | 20 | 32 |
|  |  | Scenario 4 | 30 | 40 | | 52 | | 47 | 28 | 33 | 22 | 39 |
|  |  | Scenario 5 | 44 | 48 | | 55 | | 49 | 30 | 35 | 23 | 43 |
| Vaccinated | Not infected during pandemic | Base case | 19 | 14 | | 9 | | 11 | 24 | 20 | 30 | 17 |
|  |  | Scenario 2 | 14 | 10 | | 4 | | 6 | 21 | 16 | 28 | 12 |
|  |  | Scenario 3 | 0 | 2 | | 0 | | 4 | 20 | 14 | 28 | 8 |
|  |  | Scenario 4 | 20 | 14 | | 9 | | 11 | 25 | 21 | 30 | 17 |
|  |  | Scenario 5 | 35 | 22 | | 12 | | 14 | 27 | 23 | 32 | 22 |
| Vaccinated | Infected during pandemic^a^ | Base case | 36 (34) | 29 (28) | | 16 (15) | | 25 (23) | 22 (20) | 24 (21) | 21 (19) | 25 (23) |
|  |  | Scenario 2 | 41 (41) | 33 (33) | | 21 (21) | | 30 (30) | 26 (24) | 28 (26) | 23 (21) | 29 (29) |
|  |  | Scenario 3 | 56 (56) | 41 (41) | | 25 (25) | | 33 (33) | 27 (26) | 30 (29) | 23 (22) | 34 (34) |
|  |  | Scenario 4 | 36 (34) | 29 (28) | | 16 (15) | | 25 (23) | 22 (20) | 23 (21) | 21 (19) | 25 (23) |
|  |  | Scenario 5 | 21 (18 | 21 (19) | | 13 (12) | | 22 (20) | 20 (18) | 21 (18) | 19 (18) | 20 (18) |

**^a^** The percentages between brackets give the estimated percentages of vaccinees infected before vaccination.

* outcome in the 50+ are also presented splited 50-<65 and 65+
